# Requirement of the transcription factor YB-1 for maintaining the stemness of cancer stem cells and reverting differentiated cancer cells into cancer stem cells

**DOI:** 10.1186/s13287-019-1360-4

**Published:** 2019-08-02

**Authors:** Fan Yang, Pei Cui, Yu Lu, Xiaobo Zhang

**Affiliations:** College of Life Sciences and Laboratory for Marine Biology and Biotechnology of Qingdao National Laboratory for Marine Science and Technology, Zhejiang University, Hangzhou, 310058 People’s Republic of China

**Keywords:** Cancer stem cell, Transcription factor, YB-1, Stemness

## Abstract

**Background:**

Cancer stem cells always express high levels of stemness-associated transcription factors to maintain their features. However, the regulatory mechanism of the stemness of cancer stem cells mediated by transcription factors has not been extensively explored.

**Methods:**

The YB-1 gene in cancer stem cells was knocked out by the CRISPR/Cas9 system. The YB-1 knockout cancer stem cells were transfected with a vector expressing YB-1 to rescue YB-1, and then the cell proliferation, cell cycle, apoptosis, and stemness, as well as tumorigenesis in nude mice, were assessed to examine the effect of YB-1 in cancer stem cells. The target genes of YB-1 were confirmed by CHIP-seq. The totipotency or pluripotency of differentiated cancer stem cells were detected by tumorsphere formation assay and quantitative real-time PCR.

**Results:**

The deletion of YB-1 gene inhibited the proliferation of breast cancer stem cells and melanoma stem cells, leading to cell cycle arrest and apoptosis, and induced irreversible differentiation of cancer stem cells. The tumorigenicity ability of YB-1-deleted cancer stem cells was significantly reduced in vitro and in vivo. The results of ChIP-seq showed that YB-1 maintained the stemness of cancer stem cells by promoting the expressions of stemness-associated genes (FZD-1, p21, GLP-1, GINS1, and Notch2). Furthermore, simultaneous expressions of YB-1 and the other four (SOX2, POU3F2, OCT-4, and OLIG1) or five (SOX2, SALL2, OCT-4, POU3F2, and Bmi-1) transcription factors in YB-1 knockout cancer stem cells restored the stemness of YB-1 knockout cancer stem cells.

**Conclusions:**

Our study indicated that YB-1 was required for maintaining the stemness of cancer stem cells and reverting the differentiated tumor cells into cancer stem cells.

## Introduction

Cancers composed of a heterogeneous population of cells that differ in morphology, gene expression, proliferative capacity, and invasiveness [1]. This heterogeneity may occur as a result of a small subset of cancer cells, called cancer stem cells [2, 3]. Cancer stem cells are also known as “tumor-initiating cells” or “tumorigenic cells”. In recent years, cancer stem cells have been identified and isolated from a variety of malignancies, such as colon cancer, prostate cancer, breast cancer, melanoma, multiple myeloma, and head and neck cancers [4–6]. Recent studies have provided evidence that cancer stem cells have a phenotype defined by their identity as stem cells [7]. Due to their similarity to somatic stem cells, cancer stem cells are defined as possessing the abilities of self-renewal and multipotency [8]. However, they differ from somatic stem cells. Cancer stem cells have tumorigenic activity that enables them to form tumors when transplanted into animals [2]. They are thought to be associated with the origination of tumor development, invasion, and metastasis [3]. Cancer stem cells have been implicated in the development of chemotherapeutic resistance in a number of malignances [6]. Previous studies have demonstrated that cancer stem cells are associated with treatment failure and tumor relapse in cancer [9]. These cancer stem cells are more resistant than non-stem cells to chemotherapy and radiotherapies because of their quiescent nature [10]. Cancer stem cells are reported to express high levels of anti-apoptotic proteins, to possess low levels of reactive oxygen species, and to exhibit an increased efficiency of DNA damage repair [11, 12]. Cancer stem cells have the characteristics of stem cells, but how cancer stem cells maintain stemness has not been investigated. Cancer stem cells maintain distinctive transcriptional programs that reflect their lineage and differentiation stage [13]. These transcriptional programs are driven by transcription factors via interactions with gene promoters [13]. Transcriptional and epigenetic programs can exhibit striking heterogeneity within a tumor and may distinguish cancer stem cells or other subpopulations of clinical significance [14, 15]. Cancer stem cells are reported to exhibit specific high expression of some transcription factors [16]. In this context, transcription factors may play an important role in the stemness maintenance in cancer stem cells.

Transcription factors, which bind to specific short sequences of DNA in the promoters or enhancers of their target genes [17], perform important functions in cancers. A transcription factor can be involved in regulating the expression of mediators in several potentially oncogenic downstream pathways. In cancer stem cells, many transcription factors, especially OCT4, NANOG, and SOX2, are overexpressed, a pattern common to that in early embryonic stem cells [18]. Overexpression of these genes (OCT4, NANOG, and SOX2) in human cancer stem cells is associated with tumor transformation, tumorigenicity, and tumor metastasis [19–21]. It is evident that the core stem cell factors OCT4, SOX2, and NANOG play essential roles in maintaining the pluripotency and self-renewal of embryonic stem cells, adult stem cells, and cancer stem cells [22–24]. These stem cell factors promote self-renewal of cancer stem cells by interacting with other transcription factors, such as Stat3, Hesx1, and Zic3, which can be aberrantly expressed in several types of human tumors [22–24]. From the artificial induction of combinations of “core” transcription factors, it has been thoroughly established that developmental decisions can be changed over points to produce induced pluripotent stem (iPS) cells or to induce direct lineage conversion [25]. In cancer stem cells, by the induction of a core set of neurodevelopmental transcription factors (POU3F2, SOX2, SALL2, and OLIG2) that are essential for glioblastoma propagation, differentiated glioblastoma cells can be fully reprogrammed into induced stem-like tumor-propagating cells [26]. These findings indicate that many transcription factors in cancer stem cells are closely related to stemness. However, the transcription factors necessary for stemness maintenance in cancer stem cells have not been extensively investigated.

To address this issue, the role of YB-1, a transcription factor revealed in our previous study to be associated with stemness of melanoma stem cells [27], in the maintenance of stemness of cancer stem cells was explored in this investigation. The results revealed that YB-1 was required for the maintenance of cancer stem cell stemness and the reversion of differentiated tumor cells into cancer stem cells.

## Materials and methods

### Cell culture

Human melanoma stem cells and breast cancer stem cells were sorted from MDA-MB-435 and MCF-7 cells using aldehyde dehydrogenase 1 (ALDH1), which were further confirmed by tumorsphere formation ability and in vivo tumorigenicity in our previous study [27]. Cancer stem cells were cultured in DMEM/F-12 medium (Invitrogen, USA) supplemented with 20 ng/ml epidermal growth factor (Beyotime Biotechnology, Jiangsu, China), 10 ng/ml basic fibroblast growth factor (Beyotime, China), 5 μg/ml of insulin (Beyotime, China), and 2% of B-27 (Sigma, USA) at 37 °C in a humidified atmosphere with 5% CO_2_. Melanoma and breast cancer non-stem cells were cultured in Leibovitz’s L-15 medium (Sigma, USA) supplemented with 10% fetal bovine serum (FBS) at 37 °C with 100% humidified atmosphere.

### Establishment of YB-1 knockout mutant of cancer stem cells

To knock out *YB-1* gene in cancer stem cells, a guide RNA (gRNA) (5′-GGGGCG GCGGGGGGGGCGGC-3′) was cloned into pHBCas9/gRNA-Pure vector (Hanheng Biotechnology, China). Then, the plasmid was transfected into melanoma or breast cancer stem cells using Lipofectamine 2000 (Invitrogen, USA). To evaluate the gene editing activity of gRNA, the genomic DNA of gRNA-transfected cells was extracted and the *YB-1* gene was amplified using sequence-specific primers (Table 1), followed by digestion with T7 endonuclease 1 (T7E1) (New England Biolabs, USA) at 37 °C for 30 min. The digested products were analyzed with agarose gel electrophoresis. Subsequently, the cells were cultured in the medium containing 0.5 μg/ml puromycin for 2 days. Single colony was selected, passaged, and genotyped. The *YB-1* knockout mutant of melanoma stem cells (MDA-MB-435^YB-1−/−^) or breast cancer stem cells (MCF-7^YB-1−/−^) was confirmed by DNA sequencing and Western blot with YB-1-specific antibody.

**Table 1:** The sequences of primers used in the study

YB-1	F: 5′-AGGCAGGA ACGGTTGTAGGT-3′ R: 5′-CCTTGTTCTCCTGCACCCTG-3′
GAPDH	F: 5′-GGTATCGTGGAAGGACTCATGAC-3′ R: 5′-ATGCCAGTGAGCTTCCCGTT CAG-3′
ALDH1	F: 5′-TTACCTGTCCTACTCACCGA-3′ R: 5′-CTCCTTATCTCCT TCTTCTACCT-3′
ABCG2	F: 5′-GGCCTCAGGAAGACTTATGT-3′ R: 5′-AAGGA GGTGGTGTAGCTGAT-3′
OCT-4	F: 5′-GAGCAAAACCCGGAGGAGT-3′ R: 5′-T TCTCTTTCGGGCCTGCAC-3′
Nanog	F: 5′-GCTTGCCTTGCTTTGAAGCA-3′ R: 5′-TTCTTGACTGGGACCTTGTC-3′
CDH1	F: 5′-CAAATCCAACAAAGACAAAG AAGGC-3′ R: 5′-ACACAGCGTGAGAGAAGAGAGT-3′
DSP	F: 5′-GTTTTGGGG CAGGTCAGGATT-3′ R: 5′-GGGAGGATAAGCACCGAAGAA-3′
ZO-1	F: 5′-AGC CATTCCCGAAGGAGTTGAG-3′ R: 5′-ATCACAGTGTGGTAAGCGCAGC-3′
mda-5	F: 5′-CATTAACTGTCTCATGTTCGA-3′ R: 5′-ATTGTTATCCGTTATGGT CTC-3′
mda-6	F: 5′-AGCGACCTTCCTCATCCACC-3′ R: 5′-AAGACAACTAC TCCCAGCCCCATA-3′
mda-7	F: 5′-CGGAGAGCATTCAAACAG-3′ R: 5′-GACA CAGGGAACAAACCA-3′
AP-1	F: 5′-CCCAGTGTTGTTTGTAAATAAGAGA-3′ R: 5′-CAGAAAAGAGGTTAGGGGAGTA-3′
FZD1	F: 5′-GCACTGACCAAAT GCCAATCC-3′ R: 5′-TGTGAGCCGACCAAGGTGTAT-3′
p21	F: 5′-AGCGACC TTCCTCATCCACC-3′ R: 5′-AAGACAACTACTCCCAGCCCCATA-3′
GLP-1	F: 5′-ATCTGCATCGTGGTATCCAAACTGA-3′ R: 5′-CGTGCTCGTCCATCACA AAGGT-3′
GINS1	F: 5′-CCGAAGCAAGCGGTCATACAG-3′ R: 5′-TGCCTTCA ACGAGGATGGACT-3′
Notch2	F: 5′-CCGTGTTGACTTCTGCTCTCTC-3′ R: 5′-CTACTACCCTTGGCATCCTTTG-3′
OLIG1	F: 5′-GAGGAGGAGGAAGTGGAG GAG-3′ R: 5′-CCCAGATGTACTATGCGGTTTC-3′
OLIG2	F: 5′-CGGCTGTTG ATCTTGAGACGC-3′ R: 5′-CTGGGGACAAGCTAGGAGGCA-3′
SOX8	F: 5′-CA CATCAAGACGGAGCAG-3′ R: 5′-CAGGGTAGGCACCATAGTAG-3′
ASCL1	F: 5′-GTTCAAGTCGTTGGAGTAGTT-3′ R: 5′-AAGAAGATGAGTAAGGTGGA G-3′
POU3F3	F: 5′--TCGCTCTGGACCATCTTGACA3′ R: 5′-GGCGGCTTCTAA CCCCTACCT-3′
HES6	F: 5′-AGCGACGGTAGCGTCGATGGC-3′ R: 5′-AGTGC TGGAGCTGACGGTGCG-3′
POU3F2	F: 5′-ACCTCGATGGAGGTCCGCTTT-3′ R: 5′-CTCTGGGCACCCTGTATGGCA-3′
SOX21	F: 5′-GCCATTTTGGAGCCC AGGTCG −3′ R: 5′-TGAGTCGCTGCTCGCCAATCC-3′
HEY2	F: 5′-AAAAGCAG TTGGCACAAGTCT-3′ R: 5′-ATGGCAAGAAAGAAAAGGAGA-3′
SOX5	F: 5′-T GTGAATGCTGGTAGGAGATA-3′ R: 5′-GTAGTGACCCTTACCCTGTTC-3′
RFX4	F: 5′-CGCAAGTTTTCTGGGAGGTCG-3′ R: 5′-ACGGTGGTGAACATTG TCGGC-3′
Klf15	F: 5′-AGAAACTCTTCAATCTCCTCC-3′ R: 5′-CAGCATCTT GGACTTCCTATT-3′
CITED1	F: 5′-ACTGCTTTGCGATCTTTCACC-3′ R: 5′-CC GCCAATTTATCCAACTTCT-3′
LHX2	F: 5′-AGGGAAGACCCAGAGGGTTGG-3′ R: 5′-CGCTCGGGACTTGGTTTATCA-3′
VAX2	F: 5′-GTTGAGGCGTGGGGAGG AGTT-3′ R: 5′-CCGCACCAAGCAGAAGAAAGA-3′
MYCL1	F: 5′-GGACTGG GCAGCCTCACTTTC-3′ R: 5′-CCACATCTCCATCCATCAGCAAC-3′
SALL2	F: 5′-CTTCTCCAAGGGACCCATCAC-3′ R: 5′-CCAAGCACCACGGGACTACT G-3′
SOX1	F: 5′-CGAGTTGTGCATCTTGGGGTT-3′ R: 5′-ACAGCATGATGAT GGAGACCGAC-3′
SOX2	F: 5′-AAAATCCCATCACCCACAGCAA-3′ R: 5′-AAA ATAGTCCCCCAAAAAGAAGTCC-3′
Bmi-1	F: 5′-CCCTCCACCTCTTCTTGTT TGC-3′ R: 5′-ATGACCCATTTACTGATGATTTTCG-3′
SALL4	F: 5′-TCCGCACA GCATTTCTCACAG-3′ R: 5′-AAACCCCAGCACATCAACTCG-3′
MYC	F: 5′-CG TCCTCGGATTCTCTGCTC-3′ R: 5′-CGATTTCTTCCTCATCTTCTTGTTC-3′
TCF3	F: 5′-CAGGTGGTCTTCTATCTTACTCT-3′ R: 5′-CTCAAGCAATAACTTCTCGTC-3′
ZFP57	F: 5′-CCAGCCATAGTGGGGACATCA-3′ R: 5′-GGAGGGGCTATAAAGGCAAGG-3′
FZD1 promoter	F: 5′- CGAGCTCTCGCTCCCTCTCCTCTGCCT-3′ R: 5′-CCCTCGAGGCAATCAAA TACTTTAAAGC-3′
p21 promoter	F: 5′-CGAGCTCTGGGACATGTTCCTGACGGC-3′ R: 5′- CCCTCGAGCTCAGTGTGGCCAAAGGATC-3′
GLP-1 promoter	F: 5′-CGAGCTCTCCCGG GCTGGTGGCGGGCG-3′ R: 5′-CCCTCGAGAAATGACTCCAATAATTATT-3′
GINS1 promoter	F: 5′-CGAGCTCTGCACGCCCCGCAGCTTCCT-3′ R: 5′-CCCTCGAGCGC CTCAGTCTCCCAGTGTG-3′
Notch2 promoter	F: 5′-CGAGCTCCCTGTGCACACTTTTTAT AA-3′ R: 5′-CCCTCGAGAGTGTGGGGACCTCTGTGTA-3′

### Western blot

The proteins were separated using 12% SDS-PAGE and then transferred to a polyvinylidene fluoride (PVDF) membrane. The membrane was blocked with triethanolamine buffered saline solution (TBS) containing 5% skim milk. Subsequently, the membrane was incubated with the antibody against YB-1 or β-tubulin overnight, followed by incubation with the alkaline phosphatase-conjugated secondary antibody (Roche, Switzerland) for 2 h at room temperature. After rinsing, the membrane was detected with BCIP/NBT substrate (Sangon Biotech, Shanghai, China).

### Expressions of proteins in YB-1 knockout cancer stem cells

To express an exogenous protein (YB-1, SOX2, POU3F2, OCT-4, SALL2, Bmi-1, or OLIG1) in YB-1 knockout cancer stem cells, the protein-coding gene was cloned into pcDNA3.1(+) plasmid (Invitrogen, USA). Then, the cells were transfected with 150 nM of recombinant plasmid using Lipofectamine 2000 (Invitrogen, USA).

### Cell viability analysis

Cell viability analysis was conducted with MTS [3-(4, 5-dimethylthiazol-2-yl) -5-(3-carboxymethoxyphenyl)-2-(4-sulfophenyl)-2H-tetrazolium, inner salt] assay (Promega, USA). Cells were seeded onto a 96-well plate with 100 μl of culture medium and 20 μl of MTS reagent. Then, the plate was incubated for 1.5 h at 37 °C in a humidified incubator containing 5% CO_2_. The absorbance was recorded at 450 nm.

To examine the change of cell number, 1 × 10^4^ cells were seeded onto a 6-well plate with 1 ml of culture medium. After culture for different time at 37 °C in a humidified incubator containing 5% CO_2_, the cell number was counted by Scepter™ 2.0 Cell counter. All experiments were repeated three times.

### Cell cycle analysis

Cell cycle analysis was conducted with flow cytometry. After the cell samples were fixed in ice-cold ethanol for 4 h, the cells were incubated with DNase-free RNase A (20 μg/ml) for 30 min. Then, the cells were centrifuged at 500×*g* for 5 min and stained with propidium iodide (PI) (50 μg/ml). The fluorescence intensity of 1 × 10^4^ cells was measured with a flow cytometer at an excitation wavelength of 488 nm.

### Analysis of caspase 3/7 activity

The Caspase-Glo 3/7 assay (Promega, USA) was used to evaluate the activity of caspase 3/7 according to the manufacturer’s protocol. Cells at a density of 1 × 10^4^/well were plated in a 96-well plate and cultured for 48 h. Then, 100 μl of Caspase-Glo 3/7 reagent (Promega, USA) was added. After incubation in the dark at room temperature for 30 min, the luminescence of cells was measured by a microplate reader.

### Apoptosis detection with Annexin V

The FITC-Annexin V apoptosis detection kit I (Becton, Dickinson and Company, USA) was used to examine apoptosis. Cells were harvested and rinsed with cold phosphate-buffered saline (PBS) and then resuspended in 1× annexin binding buffer at 1 × 10^6^ cells/ml. Subsequently, 5 μl of Alexa Fluor488 Annexin V and 0.1 μg of PI were added to the cells. After incubation at room temperature for 15 min in the dark, 400 μl of 1 × annexin binding buffer was added to the sample. The sample was analyzed with a flow cytometer at an excitation of 575 nm.

### Tumorsphere formation assay

Tumorsphere formation assay was conducted under non-adherent and serum-free conditions. The cells were suspended in DMEM/F-12 medium (Invitrogen, USA). Then, a single cell was plated into an ultralow adherent 96-well plate and cultured in DMEM/F-12 medium supplemented with 20 ng/ml epidermal growth factor (Beyotime, China), 10 ng/ml basic fibroblast growth factor (Beyotime), 5 μg/ml of insulin (Beyotime), and 2% of B27 (Sigma, USA). The cells were cultured for 15 days and examined under a light microscope every 5 days.

### Quantification of mRNA with real-time PCR

Total RNAs were extracted using an RNA isolation kit (Ambion). The reverse transcription reaction was conducted using PrimeScript™ RT Reagent Kit (Takara, Japan). Quantitative real-time PCR was performed with 2× TaqMan Premix Ex Taq (Takara, Japan) to detect stemness genes (OCT-4, Nanog, ALDH1, and ABCG2), differentiation genes (CDH1, DSP, ZO-1, mda-5, mda-6, mda-7, and AP-1), YB-1 target genes (FZD1, p21, GLP-1, GINS1, and Notch2), and stemness-associated transcription factors (RFX4, SOX5, SOX21, MYCL1, SOX2, POU3F2, SOX1, LHX2, VAX2, SALL2, OLIG1, SOX8, ASCL1, HES6, OLIG2, CITED1, HEY2, Klf 15, Bmi-1, SALL4, MYC, ZFP57, POU3F3, and TCF3) using gene-specific primers (Table 1). PCR reaction mixture (10 μl) contained cDNA, primer pairs, and the SYBR Green PCR Master Mix (Takara). The thermal cycle conditions included maintaining the reaction at 95 °C for 30 s, and then alternating for 40 cycles between 95 °C for 5 s and 60 °C for 30 s. GAPDH was included for normalization. The 2^−(ΔΔCt)^ method was used to calculate the relative fold change of mRNA expression.

### Chromatin immunoprecipitation (ChIP)

MDA-MB-435 melanoma stem cells were cross-linked firstly with 1% paraformaldehyde for 10 min. After washes with cold PBS, the cells were incubated with 0.125 M glycine to terminate the cross-linking. Then, the cells were lysed by lysis buffer (5 mM piperazine-1, 4-bisethanesulfonic acid, 85 mM KCl, and 0.5% Nonidet P-40), followed by incubation for 30 min on ice. After centrifugation at 3000×*g*, the nuclei of cells were resuspended in 250 μl of sonication buffer (1% sodium dodecyl sulfate, 10 mM ethylenediaminetetraacetic acid, 50 mM Tris-HCl) and sonicated to shear the DNA to a size range of 100–700 bp. Then, 50 μl of protein A agarose beads (Thermo Fisher Scientific, USA) was added to the mixture. After washes with cold PBS, the mixture was incubated with YB-1-specific antibody or control immunoglobulin G (IgG) overnight at 4 °C. Subsequently, the beads were eluted with elution buffer (100 mM NaHCO_3_ and 1% SDS). The eluate was incubated with 5 M NaCl and RNase A at 65 °C for 5 h and then with a buffer (0.5 M EDTA, 1 M Tris-HCL, proteinase K, pH 8.0) at 42 °C for 2 h, followed by DNA extraction with phenol-chloroform-isoamyl alcohol. After centrifugation at 11,000×*g* for 10 min, the upper aqueous phase was subjected to DNA precipitation with a solution (3 M NaAc, 20 μg/μl of glycogen, ethanol, pH 6.5) by incubation overnight at − 20 °C. The mixture was centrifuged at 13,000×*g* for 30 min (4 °C). The DNA was resuspended in distilled water and subjected to sequencing.

### Electrophoretic mobility shift assay (EMSA)

EMSA was performed to evaluate the binding of YB-1 protein with DNA. The YB-1 protein was purified and incubated with a DNA at different concentrations. After incubation in the reaction buffer (0.1 M KCl, 1 mM dithiothreitol, 1 mM MgCl_2_, 10 mM HEPES, pH 7.6) at 37 °C for 30 min, the mixture was separated by 1% agarose gel electrophoresis at 100 V for 25 min and then stained with ethidium bromide to examine DNA.

### Promoter activity analysis

The promoter sequence of YB-1 target gene was cloned into pGL3 firefly luciferase reporter vector (Promega, USA) with sequence-specific primers (Table 1). Then, the recombinant pGL3 was co-transfected into melanoma stem cells or breast cancer stem cells with pRL-TK renilla luciferase reporter vector (Promega, USA). The activities of firefly luciferase of pGL3 and renilla luciferase of pRL-TK were determined following the dual-luciferase reporter assay protocol recommended by Promega. The cells were washed with PBS and then lysed with passive lysis buffer (Promega, USA). Subsequently, the cell lysate was subjected to the detection of firefly luciferase activity (M1), followed by the examination of renilla luciferase activity (M2). The promoter activity was calculated according to the following formula: Promoter Activity = M1/M2.

### Tumorigenicity in nude mice

The nonobese diabetic/severe combined immunodeficient (NOD/SCID) female mice weighing ~ 25 g and aging ~ 5 weeks were used for the in vivo experiments. YB-1 knockout melanoma stem cells and wide-type melanoma stem cells were collected at 5 × 10^5^ cells/ml in physiological saline. Matrigel (Becton, Dickinson and Company, USA) was added to the cell suspension at a ratio of 1:2. Subsequently 200 μl of the cell suspension was subcutaneously injected into mice to induce tumor growth. The tumor sizes were measured by a caliper every 5 days and tumor volume was calculated as (length × width × width)/2. Forty-five days later, the mice were sacrificed by breaking neck and the solid tumors were collected by decollement using surgical scissors. The tumor sizes and weights were examined. Animal experiments were approved by The Animal Experiment Center of Zhejiang University, China. All the methods were carried out in accordance with the approved guidelines.

### Statistical analysis

All numerical data were presented as mean ± standard deviation. The data were processed using one-way analysis of variation (ANOVA) and Student’s *t* test was employed to assess the significant difference. All assays were biologically repeated for three times.

## Results

### Generation of YB-1 knockout cancer stem cells using the CRISPR/Cas9 system

**Fig. 1:**
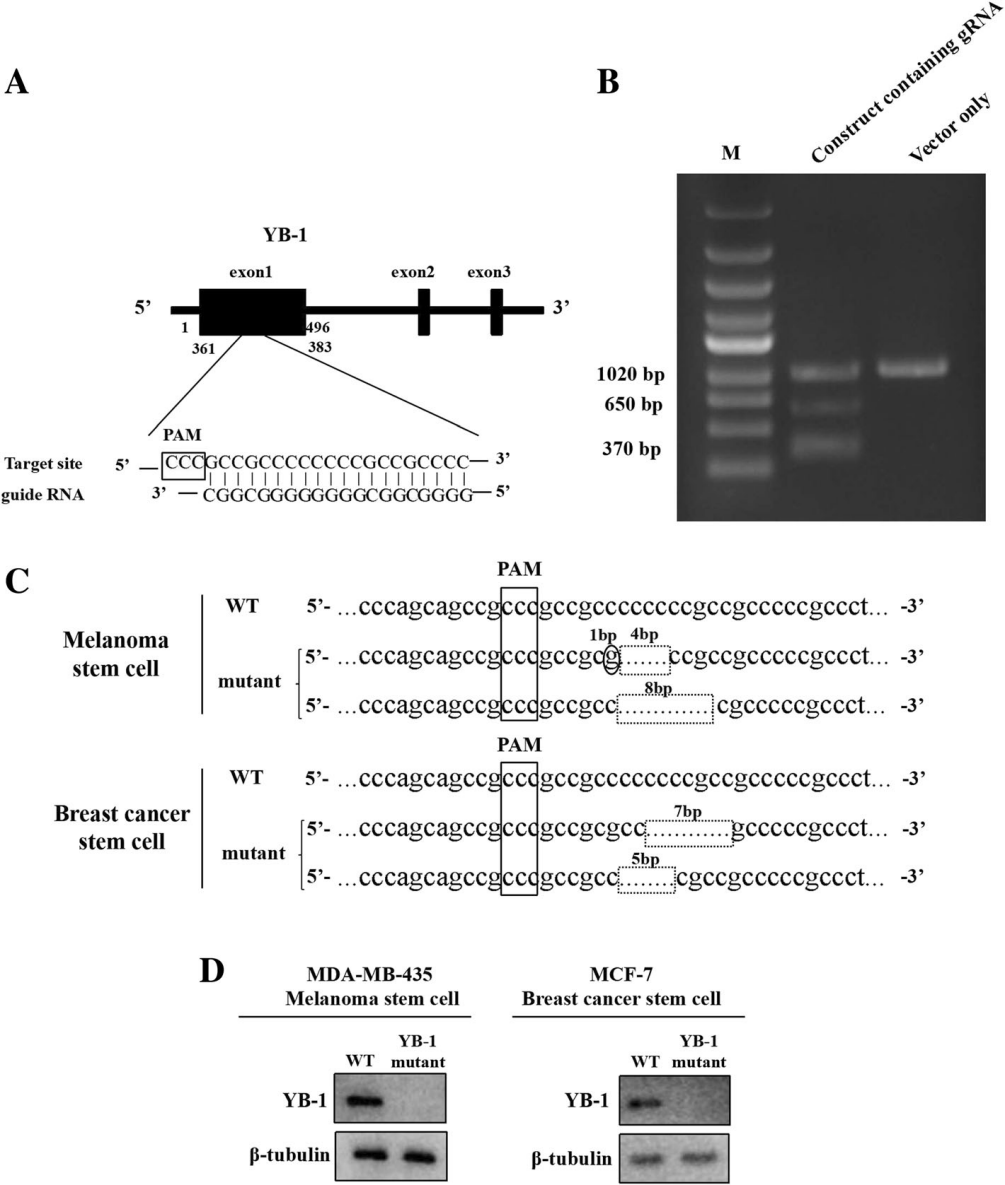
Generation of YB-1 knockout cancer stem cells using the CRISPR/Cas9 system. **a** Schematic representation of the guide RNA targeting YB-1 gene. The sequence of PAM (protospacer adjacent motif) was boxed. **b** T7 endonuclease 1 (T7E1) assay. Melanoma stem cells were transfected with pHBCas9/gRNA-Pure plasmid containing YB-1 gRNA. As a control, the vector only (without YB-1 gRNA) was included in the transfection. The DNA containing target site of YB-1 gRNA was amplified by PCR using the genomic DNA extracted from the transfected cells. The PCR product was digested with T7E1, followed by agarose gel electrophoresis. The experiments were biologically repeated for three times. M, DNA marker. **c** The sequencing of YB-1 mutant of cancer stem cells. The YB-1 gene was amplified from YB-1-mutated melanoma stem cells (MDA-MB-435) or breast cancer stem cells (MCF-7) and then sequenced. The sequences of two YB-1 alleles of mutants were indicated. The deletion mutations were shown with dashed boxes and the dot mutation was circled. **d** Western blot analysis of YB-1 in YB-1 mutants of cancer stem cells. β-tubulin was used as a control. The representative images of three experiments were shown

To evaluate the role of YB-1 in cancer stem cells, the *YB-1* gene was knocked out in melanoma stem cells (MDA-MB-435) and breast cancer stem cells (MCF-7) using the CRISPR/Cas9 system. A guide RNA (gRNA) was designed, located at 361–383 bp of YB-1 exon1 (Fig. 1a). The data demonstrated that the amplified DNA from the gRNA-transfected cells was cleaved into two bands by the T7E1 enzyme (Fig. 1b). In contrast, there was no cleaved band for control cells (Fig. 1b). These results indicated that the YB-1 gRNA was introduced into the genome of cancer stem cells. After the isolation of single cells from YB-1 gRNA-transfected cancer stem cells, the *YB-1* gene from the YB-1-mutated cancer stem cells was sequenced. The results indicated that the two alleles of the *YB-1* gene were mutated in cancer stem cells (Fig. 1c).

To confirm the YB-1 mutants of melanoma and breast cancer stem cells, YB-1 expression in the mutants was examined. Western blot analysis indicated that the YB-1 protein could not be detected in YB-1 knockout cells (Fig. 1d). The data revealed that YB-1 knockout melanoma and breast cancer stem cells were generated.

### Requirement of YB-1 for the stemness of cancer stem cells

**Fig. 2:**
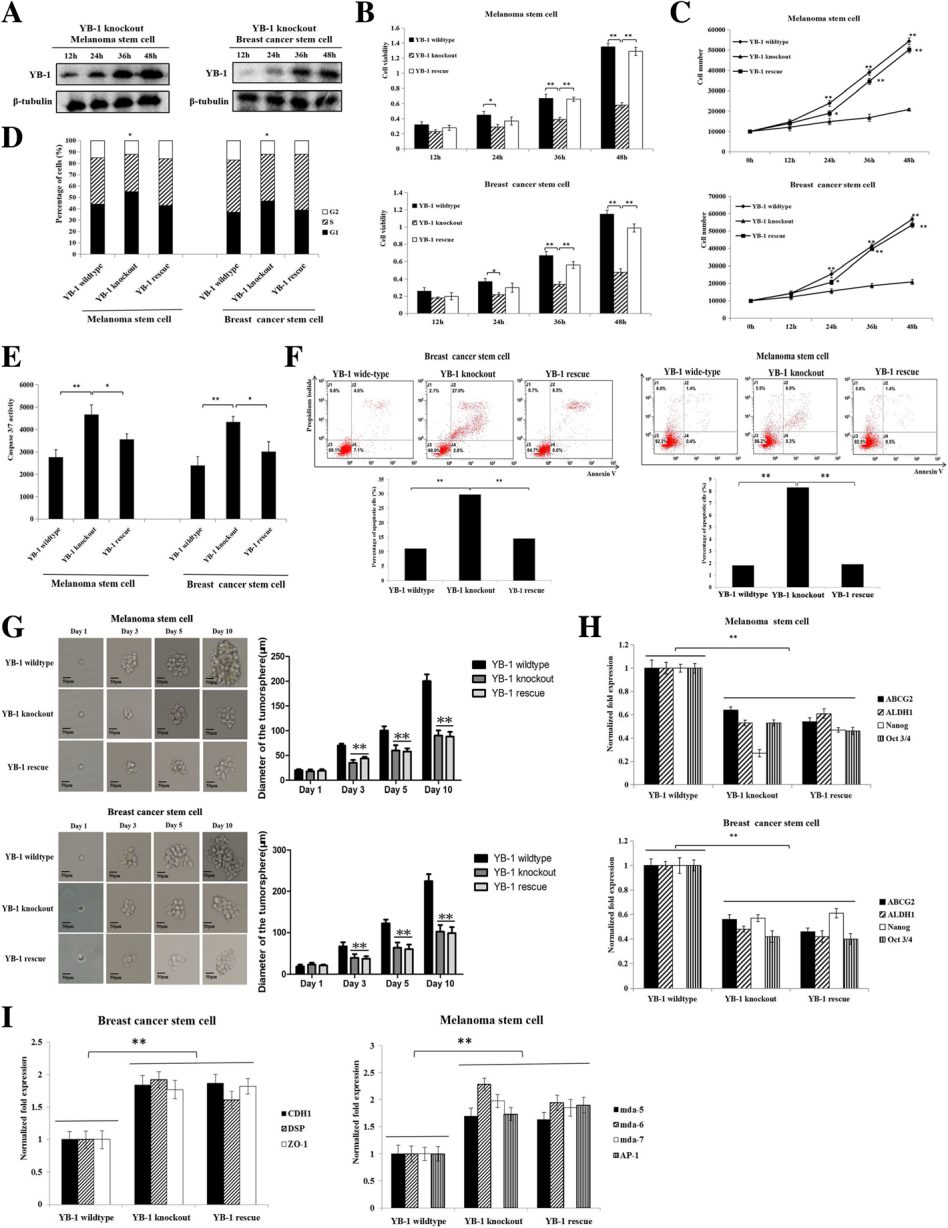
Requirement of YB-1 for the stemness of cancer stem cells. **a** The expression of YB-1 in YB-1 knockout cancer stem cells. The YB-1 knockout melanoma stem cells (MDA-MB-435) and breast cancer stem cells (MCF-7) were transfected with pcDNA-YB-1 plasmid containing YB-1 coding sequence to express YB-1. At different time after transfection, the YB-1 protein was detected with Western blot. β-tubulin was used as a control. The images were the representatives of three experiments. **b** The effect of YB-1 on cell viability of melanoma stem cells and breast cancer stem cells. YB-1 knockout, YB-1 wild-type, and YB-1-rescue cancer stem cells were seeded into a 96-well plate at 1 × 10^3^ cells/well and cultured for various time, followed by the examination of cell viability. The experiments were biologically repeated for three times. Student’s *t* test was used to assess the statistical significance of difference between treatments (*, *p* < 0.05, **, *p* < 0.01). **c** The examination of cell number. YB-1 knockout, YB-1 wild-type, and YB-1-rescue cancer stem cells were seeded into a 6-well plate at 1 × 10^4^ cells/well. At different time after treatments, the numbers of melanoma stem cells (MDA-MB-435) and breast cancer stem cells (MCF-7) were evaluated. The experiments were carried out in triplicate. The statistical significance of difference between treatments were evaluated using Student’s *t* test (*, *p* < 0.05, **, *p* < 0.01). The time point 0 h represented the number of inoculated cells. **d** The influence of YB-1 on cell cycle of cancer stem cells. YB-1 knockout, YB-1 wild-type, and YB-1-rescue cancer stem cells were cultured for 48 h and then the percentage of cells (1 × 10^4^) in the G1 phase was examined with flow cytometry. The experiments were performed in three times. Student’s *t* test was used to assess the statistical significance (*, *p* < 0.05, **, *p* < 0.01). **e** The impact of YB-1 knockout on caspase 3/7 activity of cancer stem cells. Melanoma stem cells (MDA-MB-435) and breast cancer stem cells (MCF-7) were seeded into a 96-well plate at 1 × 10^4^ cells/well subjected to the detection of caspase 3/7 activity, following culture for 48 h. The experiments were conducted for three times. Student’s *t* test was used to evaluate the statistical significance of difference between treatments (*, *p* < 0.05; **, *p* < 0.01). **f** Examination of apoptosis using Annexin V assays. Melanoma stem cells (MDA-MB-435) and breast cancer stem cells (MCF-7) were seeded into a 6-well plate at 1 × 10^5^ cells/well and culture for 48 h. Then, apoptosis of cancer stem cells were examined by flow cytometry. The experiments were biologically repeated for three times. The statistical significance of difference between treatments was assessed by Student’s *t* test (**, *p* < 0.01). **g** Influence of YB-1 knockout on the tumorsphere formation capacity of melanoma and breast cancer stem cells. At different times (1, 3, 5, and 10 days) after culture, the cells were examined under a light microscope. The statistical analysis of the diameter of tumorspheres was indicated on the right (**, *p* < 0.01). Scale bar, 50 μm. **h** Effects of YB-1 knockout on the expressions of stemness genes in MDA-MB-435 melanoma stem cells and MCF-7 breast cancer stem cells. At 48 h after cancer stem cell culture, quantitative real-time PCR was used to evaluate the expression levels of stemness genes. The experiments were performed in three times. Student’s *t* test was used to assess the statistical significance (**, *p* < 0.01). **i** Impact of YB-1 knockout on the expressions of differentiation genes in cancer stem cells. At 48 h after culture, the expression levels of differentiation genes in cancer stem cells were examined with quantitative real-time PCR. The experiments were repeated for three times. Student’s *t* test was used to assess the statistical significance of difference (**, *p* < 0.01)

To explore the role of YB-1 in cancer stem cells, the proliferation and viability of YB-1 knockout, YB-1 wild-type, or YB-1 rescue cancer stem cells was evaluated. Western blot analysis showed that YB-1 expression was rescued in YB-1 knockout melanoma stem cells and breast cancer stem cells (Fig. 2a). The results of MTS assays revealed that the viability of YB-1 knockout cancer stem cells was significantly decreased compared with that of YB-1 wild-type cancer stem cells (Fig. 2b). Rescue of YB-1 expression in YB-1 knockout cancer stem cells led to viability similar to that of YB-1 wild-type cancer stem cells (Fig. 2b). The assessment of cell number essentially generated similar results (Fig. 2c). These data indicated that YB-1 promoted the proliferation of melanoma stem cells and breast cancer stem cells.

To reveal the mechanism by which YB-1 promotes proliferation of cancer stem cells, the cell cycle of cancer stem cells was characterized by flow cytometry. The results showed that the percentage of YB-1 knockout cells in G1 phase was significantly increased compared with that of the corresponding wild-type melanoma stem cells or breast cancer stem cells (Fig. 2d). When the expression of YB-1 was rescued in YB-1 knockout cancer stem cells, the percentage of cells in G1 phase was similar to that of wild-type cancer stem cells (Fig. 2d). These findings indicated that loss of YB-1 resulted in cell cycle arrest in G1 phase.

To characterize the influence of YB-1 knockout on the apoptosis of cancer stem cells, caspase 3/7 activity of YB-1 knockout, YB-1 wild-type, or YB-1 rescue cancer stem cells was examined. The results showed that YB-1 knockout led to a significant increase in caspase 3/7 activity in melanoma stem cells and breast cancer stem cells compared with that in wild-type cells, while caspase 3/7 activity in YB-1 rescue cancer stem cells was similar to that in wild-type cells (Fig. 2e). The Annexin V assays yielded similar results (Fig. 2f). These data indicated that YB-1 knockout promoted the apoptosis of cancer stem cells.

To determine the effects of YB-1 on the stemness of cancer stem cells, the tumorsphere formation capacity of YB-1 knockout, YB-1 rescue, and YB-1 wild-type cancer stem cells was evaluated. The results indicated that YB-1 knockout significantly decreased the sphere-forming ability of melanoma stem cells and breast cancer stem cells (Fig. 2g). However, YB-1 rescue in YB-1 knockout cancer stem cells did not increase the tumorsphere formation capacity of YB-1 knockout cancer stem cells (Fig. 2g). The expression levels of stemness genes, including Oct-3/4 [18], Nanog [21], ALDH1 [28], and ABCG2 [29], were significantly downregulated in YB-1 knockout cancer stem cells compared with those in YB-1 wild-type cancer stem cells (Fig. 2h). YB-1 rescue did not promote the expression of stemness genes in YB-1 knockout cancer stem cells (Fig. 2h). These results revealed that YB-1 plays a positive role in the stemness of cancer stem cells.

To further evaluate the effects of YB-1 knockout on the differentiation of cancer stem cells, the expression levels of differentiation genes in breast cancer stem cells (CDH1, DSP, and ZO-1) [30–32] and in melanoma stem cells (mda-5, mda-6, mda-7, and AP-1) [33–36] were examined. The results showed that YB-1 knockout significantly promoted the expression of these differentiation genes (Fig. 2i), indicating that YB-1 knockout promoted the differentiation of cancer stem cells.

Taken together, these findings indicated that YB-1 was required for the stemness of melanoma and breast cancer stem cells and that YB-1 loss promoted the differentiation of cancer stem cells.

### Mechanism underlying the requirement of YB-1 for the stemness of cancer stem cells

**Fig. 3:**
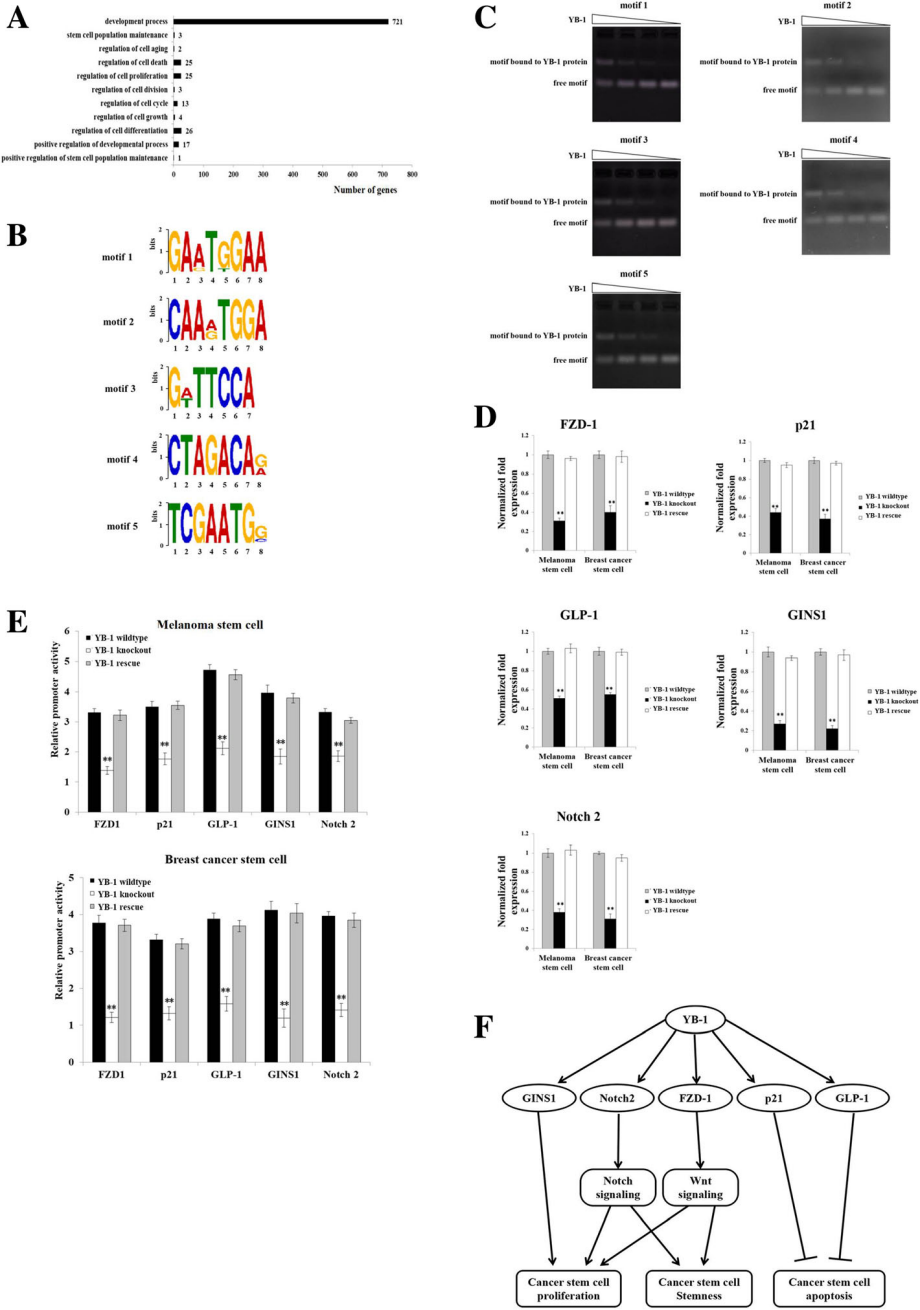
Mechanism underlying the requirement of YB-1 for the stemness of cancer stem cells. **a** Functional classification of the downstream target genes of YB-1. **b** Motifs of DNAs binding to YB-1 protein. The promoter sequences bound with the YB-1 protein could be classified into 5 motifs. **c** Direct interaction between YB-1 protein and five motifs. After incubation of a motif with the YB-1 protein, the mixture was separated by 1% agarose gel and stained with ethidium bromide to visualize the DNA. The wedges indicated the concentration gradient of YB-1 protein used. The experiments were performed in triplicate. **d** Expressions of *FZD1*, *p21*, *GLP-1*, *GINS1*, and *Notch2* genes in YB-1 knockout and rescue cancer stem cells. Quantitative real-time PCR was conducted to detect the expression levels of these genes. The experiments were repeated for three times. Student’s *t* test was used to assess the statistical significance of difference between treatments (**, *p* < 0.01). **e** Direct interaction between YB-1 and the promoters of YB-1 target genes. The plasmid containing a YB-1 target gene’s promoter and the control pRL-TK plasmid were co-transfected into YB-1 wild-type, knockout, or rescue cancer stem cells. At 36 h after transfection, the firefly luciferase activity and renilla luciferase activity were analyzed to evaluate the promoter activity. The experiments were conducted in triplicate. Student’s *t* test was used to evaluate the statistical significance (**, *p* < 0.01). **f** Proposed model of YB-1 regulating cancer stem cell proliferation and stemness

To explore the mechanism underlying the requirement of YB-1 for the stemness of cancer stem cells, the genes transcribed by the transcription factor YB-1 were analyzed by ChIP assays in melanoma stem cells using a YB-1-specific antibody. The sequencing data of ChIP products indicated that the promoter sequences were involved in the transcription of 815 genes associated with cell proliferation, apoptosis, aging, development, and stemness (Fig. 3a). These promoter sequences bound to the YB-1 protein could be classified into five motifs (motif 1, 102 genes; motif 2, 113 genes; motif 3, 87 genes; motif 4, 198 genes; and motif 5, 315 genes) (Fig. 3b). EMSA data confirmed that the YB-1 protein was directly bound to all five motifs (Fig. 3c).

To evaluate the influence of the YB-1 protein on the transcription of genes with these 5 motifs, 5 genes [motif 1, frizzled class receptor 1 (*FZD-1*); motif 2, cyclin dependent kinase inhibitor 1A (*p21*); motif 3, glucagon-like peptide-1 (*GLP-1*); motif 4, GINS complex subunit 1 (*GINS1*); motif 5, *Notch2*] were selected for analysis. The quantitative real-time PCR results showed that the mRNA levels of *FZD-1*, *p21*, *GLP-1*, *GINS1*, and *Notch2* were significantly increased with rescue of YB-1 expression in YB-1 knockout breast cancer stem cells and melanoma stem cells (Fig. 3d), suggesting that the transcription factor YB-1 was responsible for the expression of these five genes. To further confirm the involvement of YB-1 in the transcription of the *FZD-1*, *p21*, *GLP-1*, *GINS1*, and *Notch2* genes, the promoter activities of these five genes were evaluated. The results revealed that YB-1 knockout led to significant decreases in the promoter activities of the *FZD-1*, *p21*, *GLP-1*, *GINS1*, and *Notch2* genes in cancer stem cells (Fig. 3e). However, rescue of YB-1 expression resulted in significant increases in the promoter activities of the *FZD-1*, *p21*, *GLP-1*, *GINS1*, and *Notch2* genes in YB-1 knockout cancer stem cells (Fig. 3e). These data indicated that the YB-1 protein is a transcription factor responsible for the expression of 5 motif-containing genes.

According to the published data, GINS1, p21, GLP-1, Notch2, and FZD-1 play important roles in the stemness of cancer stem cells [37–42]. Combining our results and previous reports, it could be concluded that the transcription factor YB-1 promotes cancer stem cell proliferation, maintains cancer stem cell stemness, and suppresses cancer stem cell apoptosis by promoting the expression of GINS1, p21, GLP-1, Notch2, and FZD-1 (Fig. 3f).

### Role of YB-1 in the tumorigenesis of cancer stem cells in vivo

**Fig. 4:**
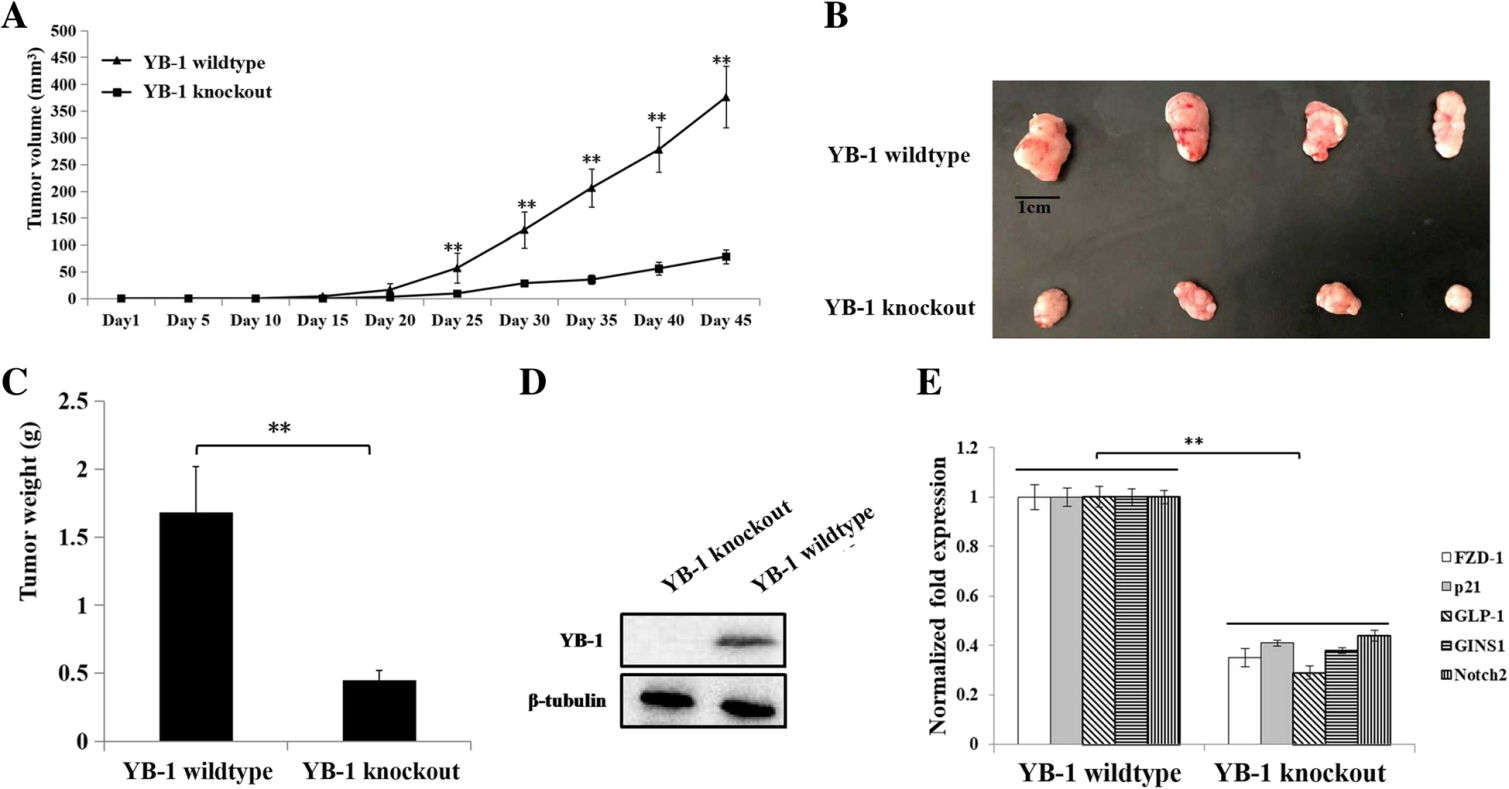
Role of YB-1 in the tumorigenesis of cancer stem cells in vivo. **a** Effects of YB-1 knockout on tumor growth in mice. YB-1 knockout melanoma stem cells and wide-type melanoma stem cells were injected into nude mice. The tumor volume in mice was measured every five days. Forty-five days later, the mice were sacrificed. The mean of four mice was indicated. The statistical significance of difference between treatments were assessed using Student’s *t* test (**, *p* < 0.01). **b** Influence of YB-1 knockout on solid tumors in mice. A solid tumor was collected from each mouse. **c** Impact of YB-1 knockout on tumor weight. The data were the means of four mice. Student’s *t* test was used to assess the statistical significance of difference (**, *p* < 0.01). **d** The YB-1 protein level in solid tumors of mice injected with YB-1 knockout melanoma stem cells or wide-type melanoma stem cells. β-tubulin was used as a control. The experiments were performed in triplicate. **e** Expressions of *FZD1*, *p21*, *GLP-1*, *GINS1*, and *Notch2* genes in solid tumors. Quantitative real-time PCR was conducted to detect the expression levels of genes. The experiments were biologically repeated for three times. Student’s *t* test was used to assess the statistical significance of difference between treatments (**, *p* < 0.01)

To evaluate the impact of YB-1 on the tumorigenesis of cancer stem cells in vivo, YB-1 knockout melanoma stem cells and wild-type melanoma stem cells were injected into nude mice. Our results indicated that tumor growth in mice injected with YB-1 knockout melanoma stem cells was significantly suppressed compared with that in control mice (Fig. 4a). The tumors in mice injected with YB-1 knockout melanoma stem cells were smaller and weighed less than those in control mice (Fig. 4b, c). In addition, Western blot data revealed that the YB-1 protein was not detected in tumors in mice injected with YB-1 knockout melanoma stem cells (Fig. 4d). These results showed that YB-1 knockout inhibited the tumorigenesis of cancer stem cells in vivo.

To explore the effects of YB-1 on the expression of stemness-related genes in vivo, the expression levels of YB-1 target genes in solid tumors were examined. The quantitative real-time PCR results showed that the mRNA levels of *FZD-1*, *p21*, *GLP-1*, *GINS1*, and *Notch2* were significantly decreased in YB-1 knockout tumors compared with those in YB-1 wild-type tumors (Fig. 4e).

These findings indicated that YB-1 promoted the expression of stemness-related genes to enhance the tumorigenesis of cancer stem cells in vivo*.*

### Requirement of YB-1 for the reversion of differentiated cancer cells into cancer stem cells

**Fig. 5:**
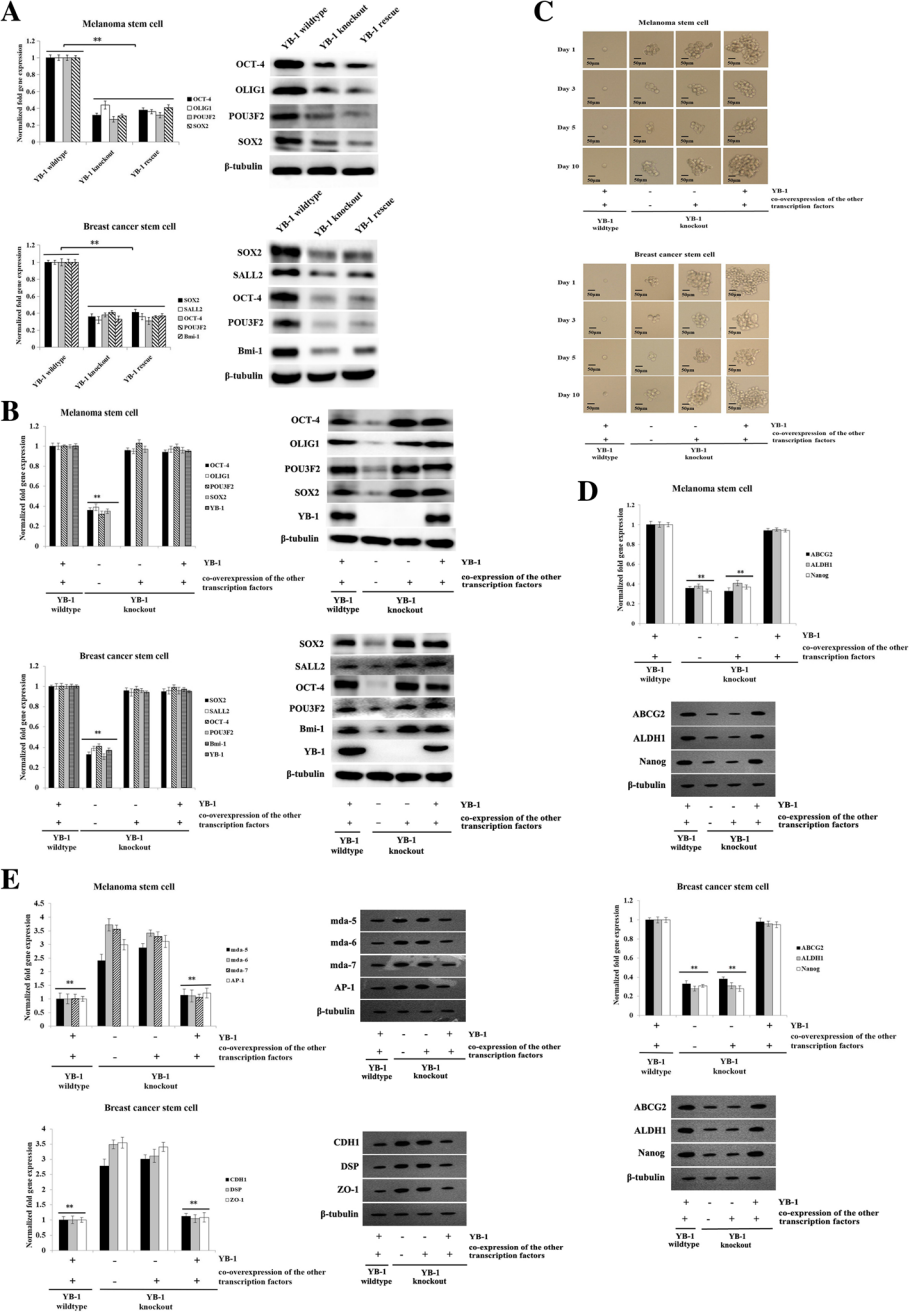
Requirement of YB-1 for the reversion of differentiated cancer cells into cancer stem cells. **a** Expressions of stemness-related transcription factors in YB-1 wild-type, YB-1 knockout, and YB-1 rescue cancer stem cells. Quantitative real-time PCR (left) and Western blot (right) were conducted to detect the expression levels of the genes. The experiments were repeated for three times. Student’s *t* test was used to assess the statistical significance of difference between treatments (**, *p* < 0.01). **b** Overexpressions of stemness-related transcription factors in YB-1 knockout cancer stem cells. The constructs of the other 4 (SOX2, POU3F2, OCT-4, and OLIG1) and 5 (SOX2, SALL2, OCT-4, POU3F2, and Bmi-1) transcription factors were transfected into the YB-1 knockout melanoma stem cells and breast cancer stem cells, respectively. At 36 h after transfection, quantitative real-time PCR (left) and Western blot (right) were performed to examine the expression levels of genes. The experiments were biologically repeated for three times (**, *p* < 0.01). **c** Influence of stemness-related transcription factors on the tumorsphere formation capacity of cancer stem cells. A single cell was plated into a 96-well plate. The cells were cultured for 10 days and examined under a light microscope at days 1, 3, 5, and 10. Scale bar, 50 μm. The experiments were repeated for three times. **d** Effects of simultaneous expressions of stemness-associated transcription factors on the expressions of stemness genes in cancer stem cells. At 36 h after transfection, quantitative real-time PCR (above) and Western blot (below) were conducted to evaluate the expression levels of stemness genes (Nanog, ALDH1, and ABCG2). The experiments were performed in triplicate (**, *p* < 0.01). **e** Impact of YB-1 rescue and simultaneous overexpressions of the other four or five transcription factors on the expression profiles of differentiation genes in YB-1 knockout melanoma stem cells and breast cancer stem cells. At 48 h after transfection, quantitative real-time PCR (left) and Western blot (right) were used to evaluate the expression levels of differentiation genes. The experiments were repeated for three times (**, *p* < 0.01)

To explore the role of YB-1 in the reversion of differentiated cancer cells into cancer stem cells, the expression of 25 stemness-related transcription factors (OCT-4, RFX4, SOX5, SOX21, MYCL1, SOX2, POU3F2, SOX1, LHX2, VAX2, SALL2, OLIG1, SOX8, ASCL1, HES6, OLIG2, CITED1, HEY2, Klf 15, Bmi-1, SALL4, MYC, ZFP57, POU3F3, and TCF3) in YB-1 wild-type, YB-1 knockout, and YB-1 rescue cancer stem cells was characterized. Quantitative real-time PCR data showed that among the 25 stemness-related transcription factors, four (SOX2, POU3F2, OCT-4, and OLIG1) and five (SOX2, SALL2, OCT-4, POU3F2, and Bmi-1) transcription factors were significantly downregulated in YB-1 knockout melanoma stem cells and breast cancer stem cells, respectively (Fig. 5a). Restoring the expression of the YB-1 protein (YB-1 rescue) in cancer stem cells yielded similar results (Fig. 5a). These data indicated that these four or five transcription factors, as well as YB-1, were essential for the reversion of differentiated cancer cells.

To revert the differentiated cancer cells into cancer stem cells, constructs expressing YB-1, SOX2, POU3F2, OCT-4, OLIG1, SALL2, and Bmi-1 were separately transfected into YB-1 knockout melanoma stem cells or breast cancer stem cells to express these proteins (Fig. 5b). The results showed that the tumorsphere formation capacity of melanoma stem cells and breast cancer stem cells was significantly decreased in the absence of YB-1, even if the other transcription factors were expressed (Fig. 5c). However, in the presence of both YB-1 and the other transcription factors, the sphere-forming ability of YB-1 knockout stem cells was comparable to that of wild-type cancer stem cells (Fig. 5c). The results of quantitative real-time PCR and Western blot showed that the expression of stemness genes (Nanog, ALDH1, and ABCG2) was significantly downregulated in YB-1 knockout melanoma or breast cancer stem cells when the other 4 (SOX2, POU3F2, OCT-4, and OLIG1) or 5 (SOX2, SALL2, OCT-4, POU3F2, and Bmi-1) transcription factors, respectively, were overexpressed (Fig. 5d). However, the simultaneous expression of YB-1 and the other transcription factors promoted the expression of stemness genes in YB-1 knockout cancer stem cells, which was consistent with the results in control cells (Fig. 5d). These data indicated that YB-1 and the other four (SOX2, POU3F2, OCT-4, and OLIG1) or five (SOX2, SALL2, OCT-4, POU3F2, and Bmi-1) transcription factors could revert the differentiated cancer cells into cancer stem cells.

To further evaluate the effects of transcription factors on the dedifferentiation of YB-1 knockout cancer stem cells, the expression levels of differentiation genes in breast cancer stem cells (CDH1, DSP, and ZO-1) and in melanoma stem cells (mda-5, mda-6, mda-7, and AP-1) were examined. The results revealed high expression levels of differentiation genes in YB-1 knockout cancer stem cells (Fig. 5e). Overexpression of the other four (SOX2, POU3F2, OCT-4, and OLIG1) or five (SOX2, SALL2, OCT-4, POU3F2, and Bmi-1) transcription factors in YB-1 knockout cancer stem cells generated similar results (Fig. 5e). However, when YB-1 expression was rescued and the other four or five transcription factors were simultaneously overexpressed in YB-1 knockout cancer stem cells, the expression profiles of differentiation genes were consistent with those in wild-type cancer stem cells (Fig. 5e). These results indicated that YB-1 was required for the reversion of differentiated cancer cells into cancer stem cells.

## Discussion

According to the cancer stem cell theory, cancer stem cells are defined as a distinct subpopulation of cancer-initiating cells and constitute a small percentage of the tumor bulk [1]. A tumor can be regarded as a cell mass with a biological hierarchy that is orchestrated by cancer stem cells [2]. The cancer stem cell population is always defined by the expression of a specific set of cell surface markers depending on the cancer of origin and is capable of both self-renewal and differentiation [43]. In addition to having the characteristics of stem cells, cancer stem cells possess some other traits, including high proliferation and strong drug resistance [5, 6]. To maintain the features of cancer stem cells, cancer stem cells constitutively express high levels of stemness-related transcription factors [18–24]. In multiple malignancies, the pluripotency and neurodevelopmental factor SRY-related HMG box-2 (SOX2) is an indispensable driver of stem-like populations [19, 22]. SOX2 expression is significantly increased in gastric cancer stem cells and inhibition of SOX2 expression in gastric cancer stem cells can suppress proliferation and induce apoptosis [19]. As the most typical stemness transcription factor, POU class 5 homeobox 1 (POU5F1, also known as OCT4) is widely expressed in almost all kinds of stem cells, including embryonic stem cells and cancer stem cells [20]. OCT-4, in cooperation with SOX2, can inhibit the differentiation of stem cells and maintain the self-renewal and stemness of stem cells [23]. During the occurrence and development of malignant tumors, OCT-4 is highly expressed in cancer stem cells, and its expression level is always positively correlated with tumor malignancy [18]. To date, however, the regulatory mechanism by which transcription factors mediate the stemness of cancer stem cells has not been extensively explored. In the present study, the results revealed that the transcription factor YB-1 could maintain the stemness of cancer stem cells by promoting the expression of stemness-related genes (*FZD-1*, *p21*, *GLP-1*, *GINS1*, and *Notch2*). As reported, the GINS1 protein is a DNA replication modulation factor that is widely expressed in stem cells to regulate proliferation and migration [37] and is closely related to the occurrence and proliferation of cancer cells [38]. The Notch2 and FZD-1 proteins, important members of the Notch and Wnt signaling pathways, respectively, in cancer stem cells, play important roles in the proliferation and stemness maintenance of cancer stem cells [39, 40]. Both the p21 and GLP-1 proteins are negative regulatory factors in apoptosis [41, 42]. In this context, the transcription factor YB-1 could promote cancer stem cell proliferation, maintain cancer stem cell stemness, and suppress cancer stem cell apoptosis by promoting the expression of GINS1, p21, GLP-1, Notch2, and FZD-1. Therefore, our study provides novel insight into the underlying mechanism of transcription factors in the stemness of cancer stem cells.

In cancer development, cancer stem cells can self-renew to form identical daughter cells by cell division and differentiation into various types of progenies [1]. These cell fate decisions are always dictated and sustained by master regulator transcription factors [15]. Normal stem cells and cancer stem cells share some core stemness transcription factors such as OCT4, SOX2, and Nanog [18]. These transcription factors bind and activate cis-regulatory elements that modulate gene transcription, thereby directing cell type-specific gene expression programs [44]. These transcription factors have vital roles in maintaining stem cell properties or regulating cell differentiation during numerous developmental processes and tumor progression [45]. In recent years, there has been accumulating evidence that some core transcription factors can induce highly differentiated cells to revert into stem cells by dedifferentiation. It is now well established that pluripotent stem cells can be directly generated from fibroblast cultures by the addition of only a few defined transcription factors (Oct3/4, Sox2, c-Myc, and Klf4) [25]. Four core transcription factors (POU3F2, SOX2, SALL2, and OLIG2) are reported to be essential for glioblastoma propagation and sufficient to fully reprogram differentiated glioblastoma cells into glioblastoma stem cells [26]. In the present study, the results showed that YB-1 knockout could induce the differentiation of cancer stem cells, leading to the decrease of sphere-forming ability and downregulation of stemness genes of cancer stem cells. However, YB-I rescue in the YB-1 knockout cancer stem cells did not reprogram the differentiated cancer stem cells into stem cells, which was consistent with the published data [25, 26]. Our results revealed that the simultaneous existence of YB-1 and the other four (SOX2, POU3F2, OCT-4, and OLIG1) or five (SOX2, SALL2, OCT-4, POU3F2, and Bmi-1) transcription factors reverted YB-1-deleted melanoma stem cells or breast cancer stem cells, respectively, into cancer stem cells. Therefore, our study showed that YB-1 is required for the reversion of differentiated cancer cells into the totipotent or pluripotent state.

## Conclusions

Collectively, our data indicated that (i) YB-1 is a key transcription factor that maintains the stemness of cancer stem cells by promoting the expression of stemness-related genes (FZD-1, p21, GLP-1, GINS1, and Notch2) and (ii) YB-1 plays a requisite role in the activation and reversion of the totipotency or pluripotency of differentiated cancer cells.

## Data Availability

The datasets used and/or analyzed during the current study are available from the corresponding author on reasonable request.

## Abbreviations

ChIP: Chromatin immunoprecipitation
EMSA: Electrophoretic mobility shift assay
FBS: Fetal bovine serum
gRNA: Guide RNA
IgG: Immunoglobulin G
iPS cells: Induced pluripotent stem cells
NOD/SCID mice: Nonobese diabetic/severe combined immunodeficient mice
PBS: Phosphate-buffered saline
PI: Propidium iodide
PVDF: Polyvinylidene fluoride
T7E1: T7 endonuclease 1
TBS: Triethanolamine buffered saline solution
